# The Homeostatic Chemokine CCL21 Predicts Mortality in Aortic Stenosis Patients and Modulates Left Ventricular Remodeling

**DOI:** 10.1371/journal.pone.0112172

**Published:** 2014-11-14

**Authors:** Alexandra Vanessa Finsen, Thor Ueland, Ivar Sjaastad, Trine Ranheim, Mohammed S. Ahmed, Christen P. Dahl, Erik T. Askevold, Svend Aakhus, Cathrine Husberg, Arnt E. Fiane, Martin Lipp, Lars Gullestad, Geir Christensen, Pål Aukrust, Arne Yndestad

**Affiliations:** 1 Research Institute of Internal Medicine, Oslo University Hospital Rikshospitalet, Oslo, Norway; 2 Department of Cardiology, Oslo University Hospital Rikshospitalet, Oslo, Norway; 3 Section of Endocrinology, Oslo University Hospital Rikshospitalet, Oslo, Norway; 4 Department of Cardiothoracic Surgery, Oslo University Hospital Rikshospitalet, Oslo, Norway; 5 Section of Clinical Immunology and Infectious Diseases, Oslo University Hospital Rikshospitalet, Oslo, Norway; 6 Institute for Surgical Research, Oslo University Hospital Rikshospitalet, Oslo, Norway; 7 Institute for Experimental Medical Research, Oslo University Hospital Ullevål, Oslo, Norway; 8 Department of Cardiology, Oslo University Hospital Ullevål, Oslo, Norway; 9 Center for Heart Failure Research, University of Oslo, Oslo, Norway; 10 K.G.Jebsen Inflammation Research Centre, University of Oslo, Oslo, Norway; 11 K.G.Jebsen Cardiac Research Centre, University of Oslo, Oslo, Norway; 12 Faculty of Medicine, University of Oslo, Oslo, Norway; 13 Department of Molecular Tumor Genetics and Immunogenetics, Max-Delbrück Center for Molecular Medicine, Berlin, Germany; 14 Department of Medicine, Lovisenberg Diakonale Hospital, Oslo, Norway; Albert Einstein College of Medicine, United States of America

## Abstract

**Background:**

CCL21 acting through CCR7, is termed a homeostatic chemokine. Based on its role in concerting immunological responses and its proposed involvement in tissue remodeling, we hypothesized that this chemokine could play a role in myocardial remodeling during left ventricular (LV) pressure overload.

**Methods and Results:**

Our main findings were: (i) Serum levels of CCL21 were markedly raised in patients with symptomatic aortic stenosis (AS, n = 136) as compared with healthy controls (n = 20). (ii) A CCL21 level in the highest tertile was independently associated with all-cause mortality in these patients. (iii) Immunostaining suggested the presence of CCR7 on macrophages, endothelial cells and fibroblasts within calcified human aortic valves. (iv). Mice exposed to LV pressure overload showed enhanced myocardial expression of CCL21 and CCR7 mRNA, and increased CCL21 protein levels. (v) CCR7^−/−^ mice subjected to three weeks of LV pressure overload had similar heart weights compared to wild type mice, but increased LV dilatation and reduced wall thickness.

**Conclusions:**

Our studies, combining experiments in clinical and experimental LV pressure overload, suggest that CCL21/CCR7 interactions might be involved in the response to pressure overload secondary to AS.

## Introduction

Aortic stenosis (AS) leads to pressure overload, myocardial hypertrophy and ultimately heart failure (HF) [1]. If left untreated, the prognosis of symptomatic AS is poor with an average survival of 2 years after onset of HF [2]. The disease progression in AS is now considered an active process and shares both risk factors and histopathological features with atherosclerosis, including lipoprotein accumulation, inflammation and remodeling of the extracellular matrix [3]–[5].

The chemokines CCL21 and CCL19, acting through their common receptor CCR7, are termed homeostatic due to their role in immune surveillance and regulation of leukocyte movement during homeostasis [6], [7]. Recent studies suggest that CCR7 and its ligands are also expressed in non-lymphoid cells such as fibroblasts, vascular smooth muscle cells, and endothelial cells, potentially being involved in vascular inflammation, cell proliferation, and matrix remodeling [8]–[10]. In line with this, the CCR7/CCL19/CCL21 dyad has also been implicated in various disorders characterized by inflammation and matrix remodeling such as atherosclerosis, rheumatoid arthritis and inflammatory bowel disease [9], [11], [12].

Recently, we demonstrated markedly increased myocardial expression of CCL21 in both clinical and experimental post-infarction HF, and we have also shown that increased serum levels of CCL21 were associated with increased total mortality in patients with acute and chronic HF [13]. Based on its essential role in concerting immunological and inflammatory responses, as well as its newly discovered involvement in tissue remodeling possibly including HF progression, we hypothesized that CCL21 could also be associated with both progression and pathogenic consequences of AS. Here, we elaborated this hypothesis by clinical studies on the association between CCL21 levels and outcome in patients with symptomatic AS. Additional studies looking for a potential role for CCL21/CCR7 in left ventricular (LV) pressure overload-induced remodeling were performed in an animal model of aortic banding, including studies in CCR7 deficient mice.

## Methods

### Ethics Statement

The clinical part of this study was approved by the Regional Health Authorities of South-Eastern Norway and conducted according to the ethical guidelines outlined in the Declaration of Helsinki for use of human tissue and subjects. Informed written consent was obtained from all subjects. All animal experiments were carried out in accordance with institutional guidelines, and conform to the Guide for the Care and Use of Laboratory Animals published by the US National Institutes of Health (NIH Publication No. 85–23, revised 1996) and was approved by the Norwegian National Animal Research Committee (permit of approval number STFDU2796).

### Patients and controls

A total of 136 patients with symptomatic AS, evaluated for aortic valve replacement (AVR) between May 2005 and January 2007 at the Department of Cardiology, Oslo University Hospital Rikshospitalet, were consecutively enrolled in the study (Table 1). All AS patients were electively evaluated for symptomatic AS at our hospital and were all in a stable clinical condition and on optimal medical treatment. Only patients with confirmed AS as assessed by echocardiography were included. Echocardiographic parameters and blood samples were obtained from all patients. Coronary angiography was performed in all patients to diagnose the presence of concomitant coronary artery disease [CAD; i.e. disease in at least one vessel (>50% narrowing of luminal diameter)]. The exclusion criteria were severe (grade III) aortic or mitral regurgitation or serum creatinine >150 µmol L^−1^. All examinations of the individual patients were conducted within a period of a few days. Of the 136 patients, 108 were scheduled for AVR, whereas surgical intervention was not performed in the remaining 28 because of co-morbidity and/or estimated high perioperative risk (n = 19), unwillingness of the patient to undergo surgery (n = 4), or because clinical benefit was uncertain owing to a mean aortic gradient <35 mmHg (n = 5). Two patients amongst those scheduled for AVR died pending surgery, thus 106 patients underwent surgical intervention. Control subjects were 20 sex- and age-matched apparently healthy individuals based on disease history and clinical examination.

**Table 1:** Patient characteristics and association (Pearsson) with plasma CCL21 levels in 136 patients with symptomatic AS.

	Total population	CCL21
Age (yrs)	74±10	0.59***
Male (%)	55	0.22*
BMI (kg/m^2^)	26.3±4.3	−0.27**
CAD (%)	43	0.21*
Current smokers (%)	33	−0.11
DM (%)	11	0.03
Hypertension (%)	25	0.05
Atrial fibrillation (%)	34	0.19*
Biochemistry		
HDL cholesterol (m*M*)†	1.6 (1.3,1.9)	0.09
LDL cholesterol(m*M*) †	3.0 (2.4,3.9)	−0.14
eGFR†	66 (52,86)	0.66***
CRP (mg/L)†	1.9 (0.9,4.4)	0.09
hsTnT (m*M*) †	14.1 (8.3,25.0) (8.3,25.0)	0.40***
Medication (%)		
ACE inhibitors	14	0.11
ARB	19	−0.07
Beta-blocker	45	0.18*
Statins	49	0.10
Warfarin	19	0.21*
Aspirin	47	−0.03
NYHA functional class	4/40/75/1	0.19*
Hemodynamics		
LVEF (%)	62±12	−0.14
CO (L/min)†	4.8 (4.2,5.6)	−0.35***
Aortic valve area (cm^2^)†	0.62 (0.50,0.80) (0.50,0.80)	−0.42***
Mean aortic gradient (mmHg)	53.5±20.2	0.16
Backscatter (dB)	18.8±5.0	0.27**
Neurohormonal		
NT-proBNP (p*M*)†	98 (42,279)	0.36***

Data are given as mean ±SD, ^†^median (1. and 3. quartiles) or percentage of total number.

BMI, Body Mass Index; CAD, coronary artery disease; DM, Diabetes Mellitus; ARB, angiotensin receptor blocker; CO, cardiac output. To convert NT-proBNP values from *pM* to *pg*/*ml* multiply by 8.47.

*p<0.05,

**p<0.01,

***p<0.001.

### Aortic valve sampling

Aortic valve specimens were obtained from patients (n = 6) undergoing elective AVR surgery. The valve cusps were excised as seen fit by the operating surgeon. Comparative immunohistochemical analyses were performed on aortic valves from persons with no medical history or macroscopic signs of heart disease (n = 4) obtained from autopsies. All aortic valve specimens were immediately immersed in formalin.

### Echocardiography

Doppler echocardiographic calculations of stroke volume and cardiac output were performed on the basis of the cross-sectional area of flow and aortic annular flow velocity data. Continuous wave Doppler from multiple positions was used to obtain the maximum aortic annular blood flow velocities, and used to calculate aortic valve area by use of the continuity equation [14]. LV ejection fraction (LVEF) was obtained by the biplane Simpson method [15]. In order to obtain a semiquantitative measure of the morphology of the stenotic aortic valve, ultrasound backscatter data analysis was performed as previously described [16]. Observers were blinded to the clinical patient status and the standard echo findings.

### Biochemistry and blood sampling

Peripheral venous blood was drawn into pyrogen-free tubes with EDTA as anticoagulant. The tubes were immediately immersed in melting ice and centrifuged within 30 minutes at 2000 *g* for 20 minutes to obtain platelet-poor plasma. All samples were stored at −80°C and had been thawed once prior to assay. N-terminal pro-brain natriuretic peptide (NT-proBNP) and C-reactive protein (CRP) were assayed on a MODULAR platform (Roche Diagnostics, Basel, Switzerland). Plasma levels of low-density lipoprotein (LDL) cholesterol, high-density lipoprotein (HDL) cholesterol and creatinine were measured enzymatically using a Roche/Hitachi 917 analyser (Roche Diagnostics, Mannheim, Germany). Estimated glomerular filtration rate (eGFR) was calculated according to the Modification of Diet in Renal Disease (MDRN) formula. Troponin T (TnT) was measured by electrochemiluminescence immunoassay (hsTnT, Elecsys Troponin T high sensitive, Roche Diagnostics). Plasma CCL21 levels were measured by enzyme immunoassay (R&D Systems, Stillwater, MN).

### Immunohistochemistry

Sections of paraffin-embedded aortic valves were deparaffinised and treated with 0.5% H_2_O_2_, followed by high-temperature unmasking in citrate buffer (pH 6), blocked with 0.5% bovine serum albumin (BSA) and then incubated with primary antibodies against human CCR7 (polyclonal ab65851, abcam, Cambridge, MA) for 1 hour at room temperature. After washing, the slides were incubated for 30 minutes with peroxidase-conjugated secondary antibody (Impress-Vector, Vector Laboratories, Burlingame, CA), rinsed, and developed with chromogen for immunoperoxidase staining (DAB Plus, Vector Laboratories) for 7 minutes. The sections were counterstained with hematoxylin. Omission of the primary antibody was used as negative control. The different cell types were identified under the microscope by a professor of pathology using typical histological characteristics of the actual cells.

### Immunofluorescence

Five micron sections of paraffin-embedded mouse hearts were deparaffinised in xylene, rehydrated in alcohol series and immersed in distilled water, followed by high-temperature antigen retrieval in citrate buffer (pH 6) and blocked with 0.5% bovine serum albumin (BSA). For immunofluorescence, the slides were stained with primary antibodies against CCR7 (NB110-55680, Novus Biologicals, Littleton, CO; 1∶100), CD31 (DIA-310, Dianova GmbH, Hamburg, Germany; 1∶100), Fibroblast Marker (sc-73355, Santa Cruz Biotechnology, San Diego, CA; 1∶100), α smooth muscle Actin (ab7817, abcam, Cambridge, UK; 1∶100) or Mac2 (CL8942AP, Cedarlane, Burlington, Canada; 1∶400) for 1 hour at room temperature and counterstained with Alexa Fluor 488 goat anti-rabbit IgG (1∶500), Alexa Fluor 568 goat anti-rat IgG (1∶500), Alexa Fluor 568 goat anti-mouse or Rat on Mouse AP Polymer kit in combination with Warp Red Chromogen kit (Biocare Medical, San Francisco, CA), respectively. The slides were mounted with SlowFade Gold antifade reagent with DAPI (Life Technologies, Carlsbad, CA). Images were taken by a Nikon Eclipse E400 microscope (Tokyo, Japan).

### Mouse model of experimental LV pressure overload

C57BL/6 mice were purchased from Møllergaard (Møllergaard, Denmark). The CCR7^−/−^ mice were constructed at the Max-Delbrück-Center for Molecular Medicine (Berlin, Germany). A 0.5 kb genomic fragment of the third exon of *CCR7* encompassing Ser-139 to Asp-309 was disrupted by insertion of the neomycin resistance gene. The herpes simplex thymidine kinase gene was then fused to the 59 end. The CCR7^−/−^ mice were backcrossed for at least 8 generations onto the C57BL/6 background and bred at the Institute for Experimental Medical Research, Oslo University Hospital Ullevål, Oslo, Norway. Banding of the ascending aorta was carried out in 7–8 week old WT and CCR7^−/−^ mice. Anaesthesia was induced with >5% isoflurane gas and <95% O_2_ in a gas chamber, followed by tracheotomy and ventilation with 2% isoflurane and 98% O_2_, with a tidal volume of 350 µl and a respiratory frequency of 160 min^−1^ on a MiniVent ventilator (Harvard Apparatus, Holliston, MA). A sternal split of the cranial 1/3 of the sternum was performed and an 8–0 silk ligature was tied around the ascending aorta and a blunted 26G needle, which was subsequently removed. Sham-operated animals underwent the same procedure without banding of the aorta. Three weeks after surgery, the animals were anesthetized and ventilated before echocardiography was carried out using a VEVO 2100 (Visualsonics, Toronto, Canada) as previously described [17]. The animals were subsequently euthanized and their hearts were removed and blotted dry. The left ventricle, right ventricular free wall, and lungs were weighed and normalized to tibia length. Only mice with a maximum flow velocity across the stricture >3.5 m/s three days after AB were included.

### Quantitative Real-Time RT-PCR

Total RNA from mouse left ventricle was extracted using TRIzol (Invitrogen, San Diego, CA), DNase treated, cleaned up using RNeasy Mini Columns (Qiagen, Hilden, Germany), and stored at −80°C. cDNA was synthesized from 1 µg RNA using High Capacity cDNA Archive Kit (Applied Biosystems, Foster City, CA). Quantification of gene expression was performed using the ABI Prism 7500 (Applied Biosystems), 5 ng cDNA, Power SYBR Green Master Mix (Applied Biosystems), and sequence-specific PCR primers were designed using the Primer Express software, version 3.0 (Applied Biosystems). Primer sequences can be provided on request. Gene expression of the housekeeping gene GAPDH was used for normalization.

### Western blotting

Total protein homogenates of LV from sham operated mice and from mice with compensated and decompensated hypertrophy due to aortic banding-induced pressure overload. Briefly, LV tissue was homogenized in T-PER Tissue protein extraction reagent (Thermo Scientific, Rockford; IL) supplemented with protease and phosphate inhibitors (Halt Protease and Phosphatase Inhibitor Cocktail; Thermo Scientific). The homogenates were cleared by centrifugation and concentrations were measured using the BCA assay (Bio-Rad, Hercules, CA). Protein homogenates were separated under denaturing conditions on 4–20% SDS-polyacrylamide gels (Mini-PROTEAN TGX Precast gels) and electroblotted on to PVDF membranes (Thermo Scientific). The membranes were blocked in Superblock T20/TBS (Thermo Scientific) and incubated with 0.1 µg/ml (diluted in Superblock T20/TBS) goat anti-mouse CCL21 antibody (AF457; R&D Systems) and subsequently a horseradish peroxidase-conjugated donkey anti-goat antibody (Santa Cruz Biotechnology, Santa Cruz, CA). CCL21 expression was detected by chemiluminescence (SuperSignal West Pico; Thermo Scientific) and the C-Digit Blot Scanner (Li-Cor). The membranes were stripped using Restore Western Blot Stripping buffer (Thermo Scientific), blocked and reprobed with 0.05 µg/ml mouse anti-GAPDH antibody (G8795, Sigma-Aldrich). Densitometrical quantification was performed using ImageJ (NIH).

### Analysis of myocardial hydroxyproline contents

Quantitative analysis of tissue contents of hydroxyproline was performed by HPLC using the AccQ-Fluor reagent kit (Waters Corporation Milford, MA, USA) essentially as previously described [18]. The relation of myocardial hydroxyproline contents to myocardial collagen has previously been reported [19].

### Zymography

Total protein homogenates (40 ug, *n* = 6) from WT and CCR7^−/−^ LV subjected to pressure-overload were mixed with 2× non-reducing SDS-PAGE sample buffer and resolved through a 10% Tris-Glycine gel with 0,1% gelatin (Novex Zymogram Gelatin Gel, Invitrogen, San Diego, CA). After electrophoresis the gels were first incubated in 100 mL Novex Zymogram Renaturing Buffer and then in 100 mL Novex Zymogram Developing Buffer (both Invitrogen, San Diego, CA) for 30 min under gentle agitation at room temperature, before incubating overnight (20–24 hours) in 100 mL Novex Zymogram Developing Buffer at 37°C. The gels were subsequently rinsed 3×5 minutes with deionized water under gentle agitation at room temperature, before they were stained in 20 mL SimplyBlue Safestain (Life Technologies AS) for one hour at room temperature under gentle agitation. Finally, the gels were rinsed 2×1 hour in deionized water, scanned with a resolution of 300 dpi and saved as TIFF images. Band intensities were measured using ImageJ (Wayne Rasband, National Institute of Mental Health).

### Statistical analysis

CCL21 levels between patients and controls were compared with the Mann-Whitney U-test. If more than three groups were compared, the Kruskal-Wallis test was used *a priori*. Associations between CCL21 levels and clinical variables (Table 1) were analyzed by linear regression on log transformed measures as necessary determined by normality assessed by the Kolmogorov–Smirnov test. The association of CCL21 with all-cause mortality was investigated by receiver-operating characteristics (ROC) and Kaplan–Meier analysis with log-rank test performed to compare the number of events in different groups (comparisons pooled over strata). Follow-up time for all-cause mortality was calculated from time of inclusion to death from any cause. P values are two-sided and considered significant when <0.05. All analyses were performed with SPSS for Windows version 15.0 (SPSS, Chicago, IL).

## Results

### Circulating CCL21 levels in patients with AS and associations with clinical features and myocardial function

**Figure 1:**
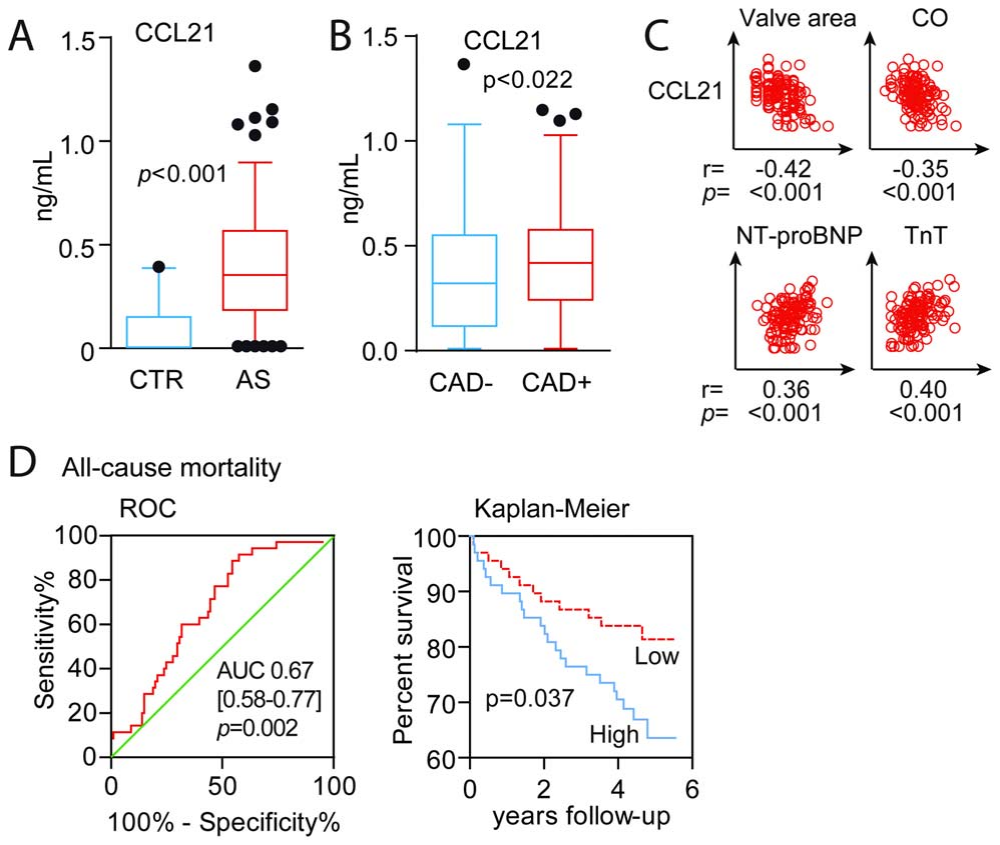
Circulating CCL21 levels in patients with AS and associations with clinical features and myocardial function. (**A**) Plasma levels of CCL21 in 136 patients with symptomatic aortic stenosis compared to healthy controls (n = 20). (**B**) Plasma levels of CCL21 in the aortic stenosis patients with and without coronary artery disease (CAD). Data in A and B are shown as a box and whisker plot with median (Q1, Q3) in the box and the whiskers representing the 5 and 95 percentile. Outliers are shown as filled circles. (**C**) Scatter plots showing associations between plasma CCL21 and aortic valve area, cardiac output, N-terminal pro-brain natriuretic peptide (NT-proBNP) and troponin T (TnT). (**D**) Association between plasma CCL21 and all-cause mortality in patients with symptomatic aortic stenosis. Receiver-operating characteristics curve analysis (area under the curve) for the predictive value of CCL21(left panel) and Kaplan–Meier curves showing the cumulative incidence of all-cause mortality according to median CCL21 (low, high) levels at enrolment (right panel).

*Figure 1A* shows that plasma CCL21 levels were markedly elevated in patients with symptomatic AS (n = 136) compared with sex- and age-matched healthy controls (n = 20). The characteristics of patients and their associations with CCL21 levels are shown in Table 1. Several notable correlations were observed including increasing CCL21 levels in relation to advancing age and declining kidney function. Patients with high CCL21 levels were also more likely to be males, to have accompanying CAD and atrial fibrillation, to use ß-blockers and warfarin and to have a lower body mass index (BMI). Although CCL21 was correlated with the presence of CAD, raised CCL21 levels were also found when these patients were excluded from the analyses (p<0.001 versus control, *Figure 1B*). Of relevance for increased LV outflow obstruction and hypertrophy in AS, elevated CCL21 was also associated with decreased aortic valve area and cardiac output as well as with increased NT-proBNP and TnT as circulating markers of hemodynamic burden and wall stretch, and myocyte injury, respectively (Table 1 and *Figure 1B*). Including all the above mentioned variables associated with CCL21 (i.e. age, gender, BMI, CAD, atrial fibrillation, eGFR, TnT, β-blocker and warfarin use, NYHA functional class, cardiac output, aortic valve area, backscatter and NT-proBNP) in a stepwise regression revealed that the strongest determinants of circulating CCL21 were (given as standardized coefficient, t and p-value) in descending order: eGFR (Beta −0.49, t = −6.11, p<0.001), TnT (Beta 0.21, t = 2.78, p = 0.007), aortic valve area (Beta −0.17, t = −2.29, p = 0.024) and backscatter (Beta = 0.14, t = 2.00, p = 0.049) with an r square of 0.57 in the final model.

We have previously showed enhanced CCL21 levels in patients with HF following MI. Although caution is needed when comparing cytokine levels between different studies, AS patients seem to have higher levels compared to the post-MI HF population (median [25/75 percetile]; 224 [81,415] pg/ml VS. 360 [186,567] pg/ml, p<0.001, post-MI HF and AS patients, respectively). These data could possibly imply that the high levels in AS do not merely reflect that these patients also have HF. However, when dividing AS patients into LVEF below and above 40% we found that those with reduced LVEF were characterized by increased CCL21 levels (median [25/75 percetile]; 339 [183,553] pg/ml VS. 553 [395,581] pg/ml, P = 0.022) suggesting that the high CCL21 levels in AS patients, at least to some degree, also are influenced by the degree of LV dysfunction in these patients.

### CCL21 is associated with all-cause mortality

During a median (Q1–Q3) follow-up of 4.6 (4.0,4.9) years, 35 patients died. CCL21 concentrations were significantly (p = 0.002) higher in decedents (0.46 ng/mL [0.33,0.65]) than in survivors (0.32 ng/mL [0.15,0.53]) and ROC analysis also demonstrated a reasonable accuracy for the prediction of all-cause mortality at follow-up (*Figure 1C, left panel*). This was confirmed in Kaplan-Meier analyses demonstrating increased mortality with above median levels of CCL21 (*Figure 1C, right panel*).

### Increased CCR7 immunoreactivity in calcified aortic valves

**Figure 2:**
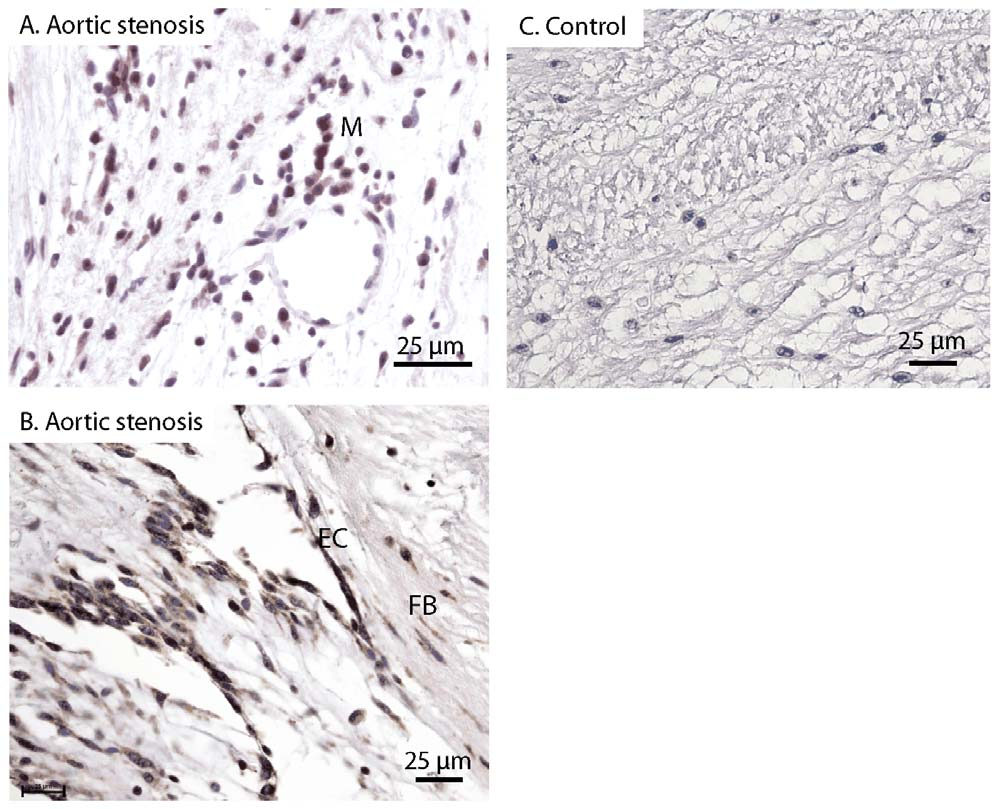
Immunohistochemical staining for CCR7. Left panels (**A** and **B**) show positive immunostaining of valves from patients with symptomatic AS. Right panel (**C**) shows negative immunostaining of valve from control. EC: Endothelial cells; M: Macrophage-like cells; FB: Fibroblasts. Representative images obtained with ×40 objective. Scale bar: 25 µm.

CCL21 mediates its effects through CCR7 and we therefore analyzed the expression of CCR7 in aortic valves from six of the patients with significant AS and 4 patients without AS (controls) by means of immunohistochemistry. In AS patients, immunostaining revealed significantly increased, although varying degrees, of positive staining of macrophage-like cells as opposed to aortic valves from control patients which did not reveal a significant amount of CCR7 positive staining of these cells (*Figure 2*). CCR7 positive endothelial and fibroblast cells were found in valves from both AS patients and controls. Despite considerable heterogeneity, overall immunostaining showed increased presence of CCR7 within calcified aortic valves.

### Myocardial CCL21 and CCR7 mRNA and CCL21 protein expression is increased in experimental LV pressure overload

**Figure 3:**
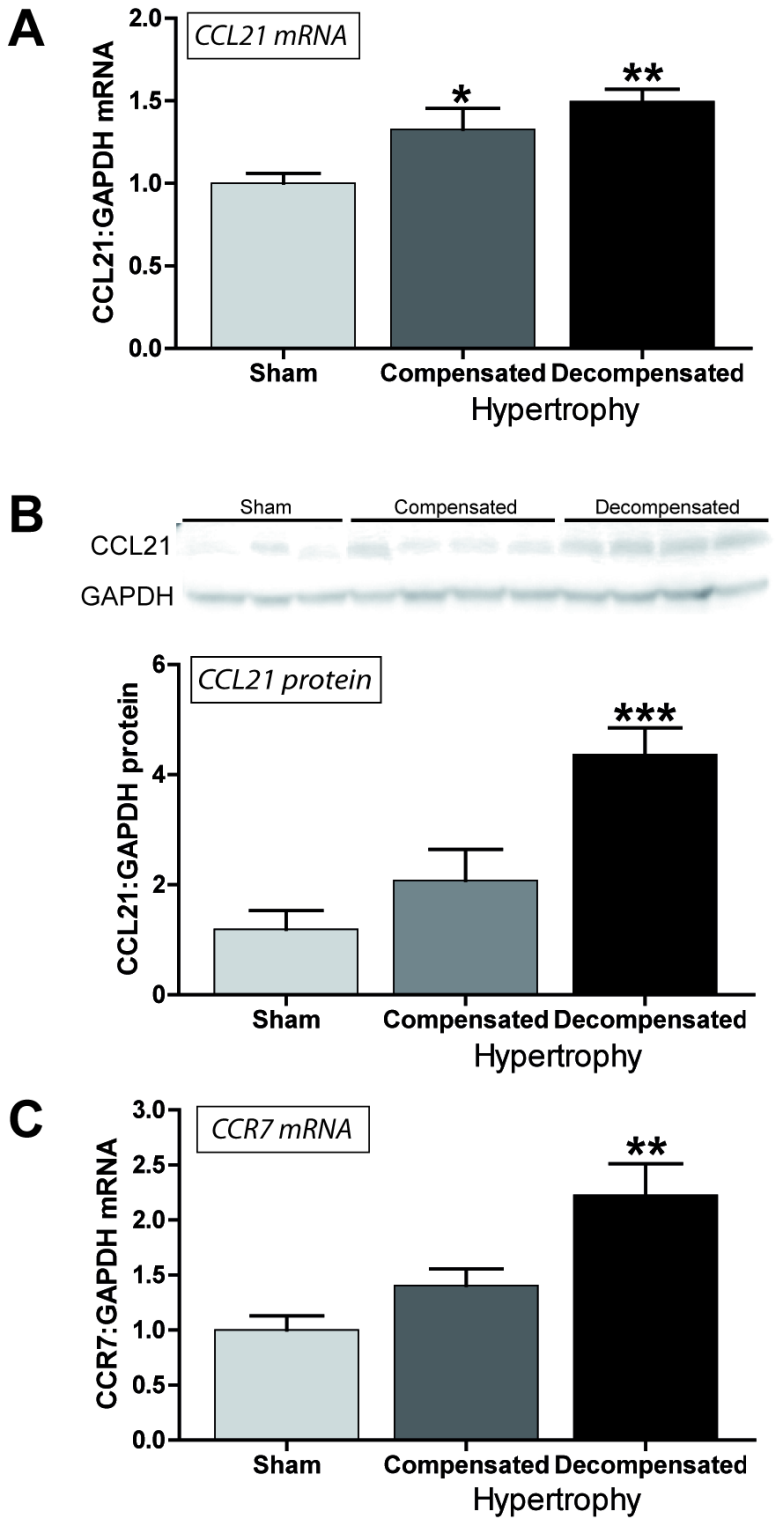
Myocardial CCL21 and CCR7 mRNA and CCL21 protein expression in experimental LV pressure overload. Left ventricular expression of (**A**) CCL21 mRNA, (**B**) CCL21 protein levels and (C) CCR7 mRNA in mice with compensated (n = 7) and de-compensated (n = 7) hypertrophy three weeks after aortic banding, compared to sham operated mice (n = 7). mRNA levels were quantified by real-time RT-PCR and are presented relative to the gene expression of GAPDH. CCL21 protein levels were quantified by Western blotting. Data are mean±SEM. *p<0.05, **p<0.01 and ***p<0.001 vs. controls/sham.

**Figure 4:**
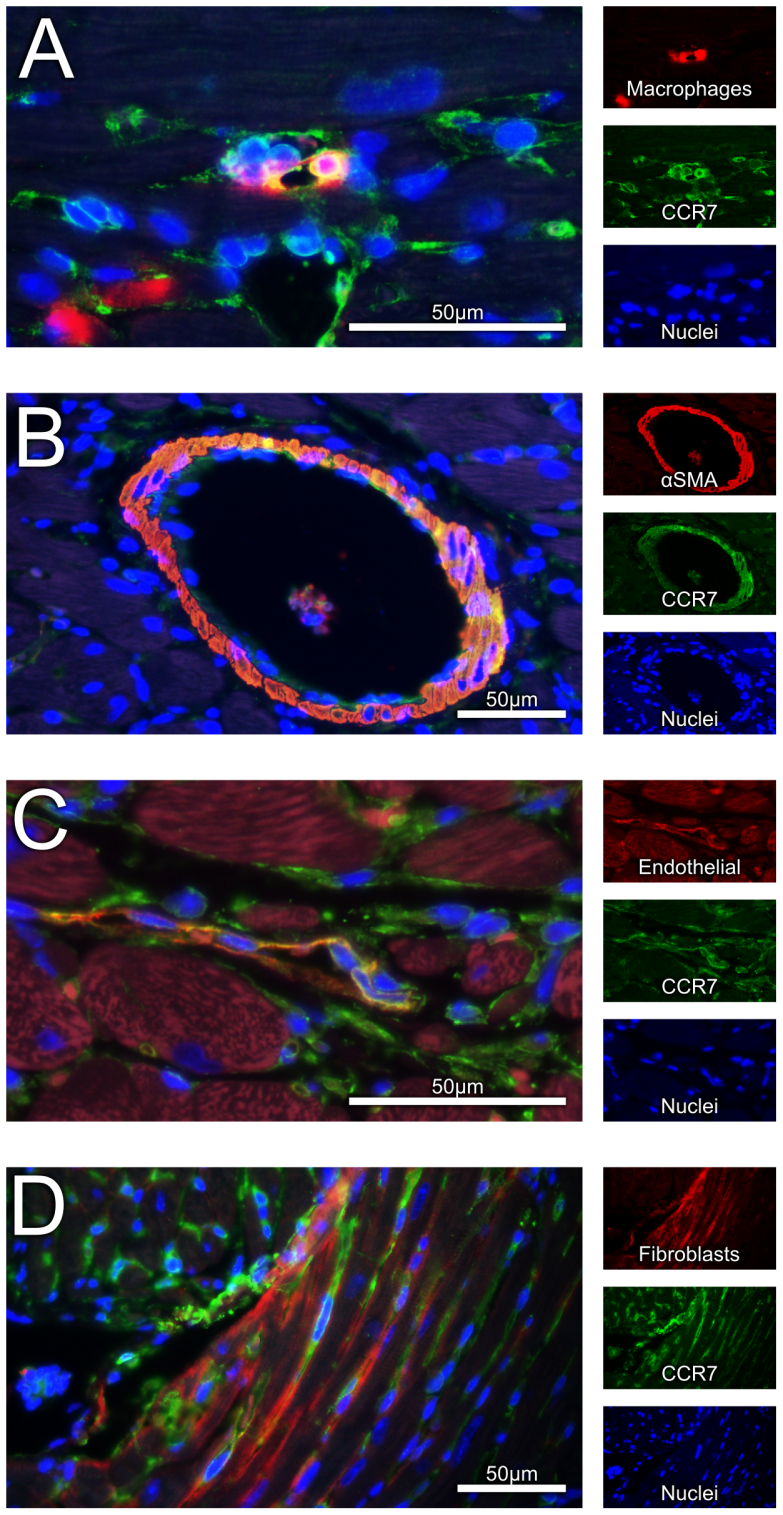
Immunofluoresence staining for CCR7. Presence of CCR7 on (**A**) macrophages, (**B**) smooth muscle cells, (**C**) endothelial cells, and not on (**D**) fibroblasts. Representative images obtained with ×40 objective. Scale bar: 50 µm.

Our findings so far suggested increased serum levels of CCL21 in symptomatic AS, which was significantly associated with markers of impaired myocardial function, degree of AS and total mortality during follow-up, and notably, an up-regulation of CCR7 in aortic valves from AS patients. To further examine the association between CCL21 and AS, we examined the myocardial expression of CCL21 and CCR7 in an experimental model of myocardial pressure overload (i.e., aorta banding, AB). As shown in *Figure 3A*, we found a significant myocardial up-regulation of CCL21 in hearts with compensated (n = 7) and in particular in de-compensated (n = 7, significantly increased wet lung weight) LV hypertrophy three weeks following AB as compared with sham operated mice (n = 7). Furthermore, Western blotting showed a stepwise increase in CCL21 protein levels, which was statistically significant in the de-compensated hypertrophic heart (*Figure 3B*). Myocardial expression of CCR7 mRNA was only increased significantly in hearts with de-compensated hypertrophy (*Figure 3C* *).* CCR7 protein levels as assessed by Western blotting were not detectable due to its low expression in murine hearts. However, by using immunofluorescent staining of pressure-overloaded hearts we demonstrated CCR7 expression in macrophages, smooth muscle cells and endothelial cells, while no expression was seen in fibroblasts (Figure 4).

### CCR7^−/−^ mice display LV dilatation in response to experimental LV pressure overload

As expected, WT mice developed myocardial hypertrophy with significant increases in septum and LV posterior wall thickness as assessed by echocardiography (*Table 2*), as well as increased LV weight (*Table 2*) three weeks after AB. AB resulted in a similar increase in LV weight in CCR7^−/−^ mice. However the CCR7 deficient mice displayed ventricular dilation in response to pressure overload, reflected in larger LV diameters, significantly thinner septum and posterior walls, as well as lower calculated relative wall thickness (*Table 2*). There were no significant changes in wet lung weights following AB in either genotype (*Table 2*) indicating compensatory hypertrophy in both genotypes. However, there was a tendency towards reduced fractional shortening in CCR7^−/−^ mice indicating mildly worsened cardiac function (*Table 2*).

**Table 2:** Animal characteristics and echocardiographic measurements 3 weeks after sham operation or aortic banding.

	Sham-Wt	Sham-CCR7^−/−^	AB-Wt	AB-CCR7^−/−^
	(n = 6–7)	(n = 6–8)	(n = 7–8)	(n = 10–11)
*Characteristics*				
BW, g	25.8±1.0	24.0±0.7	25.1±1.1	24.9±0.5
TL, mm	16.9±0.2	16.6±0.1	16.6±0.2	16.6±0.1
LVW/TL, mg/mm	4.91±0.09	4.73±0.52	7.39±0.20*	7.43±1.10*
RVW/TL, mg/mm	1.23±0.07	1,35±0.06	1.36±0.06	1.52±0.14
LW/TL, mg/mm	8.48±0.20	8.85±0.18	12.62±1.40*	12.84±2.20
*Echo-MM*				
IVSd/TL	0.05±0.00	0.06±0.00	0.08±0.00*	0.07±0.00* ^†^
LVDd/TL	0.21±0.00	0.24±0.01	0.20±0.01	0.26±0.01^†^
PWd/TL	0.06±0.00	0.06±0.00	0.08±0.00*	0.07±0.00* ^†^
LVFS, %	22±2	25±2	27±2	20±2
LAD/TL	0.08±0.00	0.08±0.01	0.10±0.01*	0.12±0.01*
*Echo-Doppler*				
HR, beats/min	383±15	403±21	409±42	440±36
Mit V_max_, m/s	0.39±0.03	0.44±0.03	0.50±0.04	0.53±0.03
Mit dec, m/s^2^	15.9±1.1	17.6±1.6	28.25±5.19*	29.86±2.72
AB V_max_, m/s			4.53±0.23	4.62±0.23
LVOT V_max_, m/s	1.12±0.07	1.51±1.13^†^	1.03±0.11	1.06±0.05*
LVOT VTI, cm	5.1±0.3	6.04±0.4	4.91±0.71	5.05±0.47
CO, ml/min	20.6±1.4	30.6±3.7^†^	28.8±6.4	30.1±4.1

Sham, sham operated group; AB, aorta banding group; WT, wild-type mice; CCR7^−/−^, CCR7 knock-out mice; BW, body weight; TL, tibia- length; LVW/TL, left ventricular weight normalized to TL; RVW/TL, right ventricular weight normalized to TL; LW/TL, lung weight normalized to TL; Echo-MM, M-mode echocardiography; IVSd/TL, interventricular septum thickness in diastole normalized to TL; LVDd/TL, left ventricular diameter in diastole normalized to TL; PWd/TL, posterior wall thickness in diastole normalized to TL; LVFS, left ventricular fractional shortening; LAD/TL, left atrial diameter normalized to TL; HR, heart rate; Mit Vmax, peak mitral flow; Mit dec, mitral deceleration velocity; AB Vmax, peak flow through aortic banding; LVOT Vmax, peak left ventricular outflow tract flow; VTI, velocity time integral; CO, cardiac output;

*p<0.05 vs sham in respective genotype group and ^†^p<0.05 vs Wt in same group. Values are means ± SEM.

### WT and CCR7^−/−^ mice demonstrate similar myocardial collagen content, as well as MMP-2 and MMP-9 activity following pressure overload

**Figure 5:**
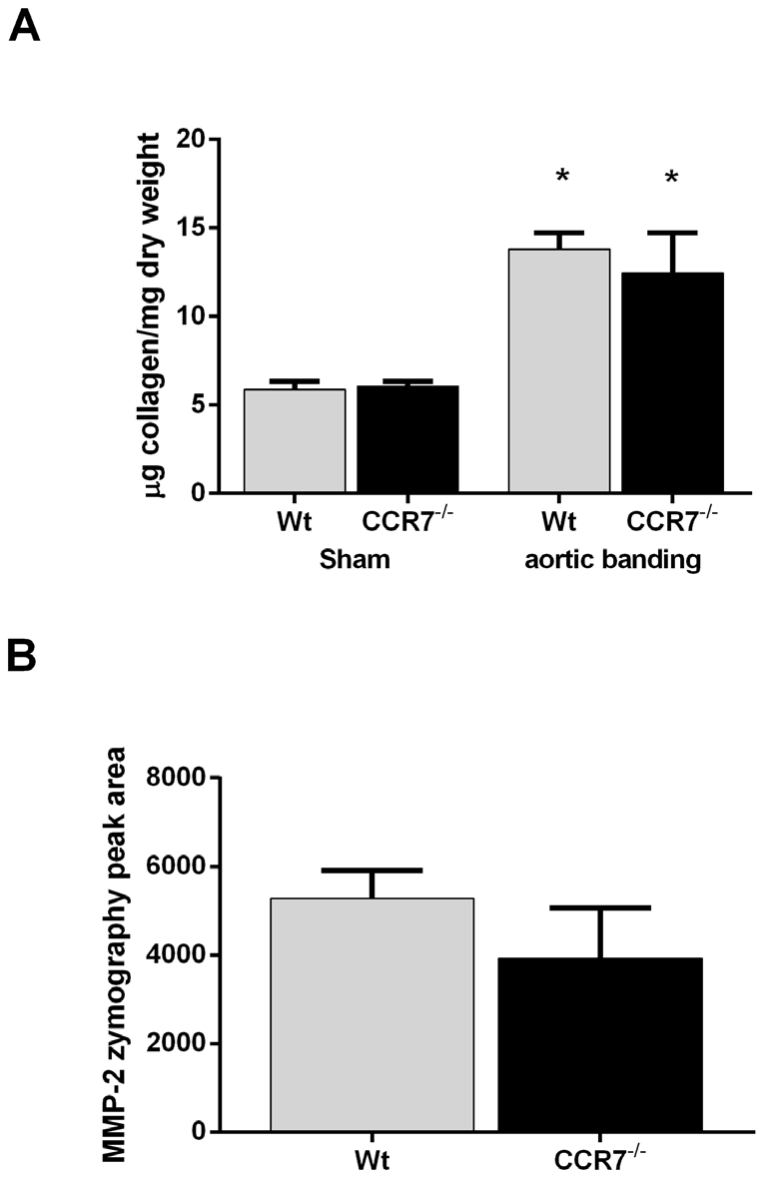
Myocardial collagen content and MMP activity following pressure overload. (**A**) Myocardial collagen content measured by quantitative analysis of tissue hydroxyproline by HPLC in left ventricles from wild type (WT) and CCR7^−/−^ mice three weeks after sham-operation and aorta banding. (**B**) Total left ventricular MMP-2 activity measured in wild type (WT) and CCR7^−/−^ mice three weeks after aorta banding by zymography. Data are mean±SEM. *p<0.05 vs. sham operated in same genotype.

In search of underlying mechanisms for the increased dilatation observed in CCR7^−/−^ mice following AB, myocardial collagen content was measured by means of quantitative analysis of hydroxyproline. As shown in *Figure 5A*, a similar and significant increase in collagen content was seen following pressure-overload in both genotypes. Furthermore, no significant difference in myocardial MMP-2 activity was detected on zymography 3 weeks after AB, when comparing the two genotypes (*Figure 5B*). MMP-9 activity was not detectable on zymography for either genotype.

## Discussion

CCR7 and its ligands have previously been related to various inflammatory disorders including atherosclerosis and have also been linked to development of HF [9], [12], [20]. Our present data suggest that this chemokine system also could be involved in the progression of AS and myocardial remodeling in response to pressure overload. For the CCR7 ligand CCL21, markedly enhanced expression was found systemically in patients with symptomatic AS, and high serum levels of CCL21 were associated with increased mortality in these patients. Moreover, increased CCR7 immunoreactivities were found in calcified aortic valves from patients undergoing AVR surgery. Finally, our studies in CCR7^−/−^ mice showed increased LV dilation in response to three weeks of pressure overload, indicating a role for CCL21/CCR7 in preserving LV geometry during cardiac stress.

The present study is, to the best of our knowledge, the first report of elevated serum levels of homeostatic chemokines in patients with symptomatic AS. We have previously shown increased CCL21 levels in CAD [9] and in acute and chronic post-infarction HF [13]. One could claim that the raised CCL21 levels in AS patients might merely reflect the presence of accompanying CAD and HF in these patients. However, raised CCL21 levels were also found when patients with CAD were excluded from the analyses. Moreover, although patients with the lowest LVEF had the highest CCL21 levels, suggesting some influence of LV dysfunction on CCL21 levels, the high levels in AS patients as compared with our previous data on CCL21 levels in post-MI HF [13], suggest that the raised levels of CCL21 in AS patients do not merely reflect accompanying HF. There was a significant association between CCL21 and backscatter as a marker of aortic valve calcification, and in particular with decreased aortic valve area. In fact, CCL21 was significantly associated with aortic valve area and backscatter also after correction for several confounders. Moreover, in the experimental model of pressure overload following AB, myocardial CCL21 mRNA expression was significantly increased in both compensated and decompensated hypertrophy, and CCL21 protein levels were significantly increased in hearts with overt HF. These findings suggest that CCL21 might have a role in the remodeling process secondary to pressure overload and not merely reflect associated conditions in these patients.

CCL21 seems primarily to be produced by stromal cells [6]. It is possible that the ability of CCL21 to predict mortality may reflect its regulation in stromal-related cells within the myocardium. In the present study we found enhanced myocardial expression of CCL21 and CCR7 during experimental pressure overload and increased expression of CCR7 within aortic valves of AS patients. While these data show a myocardial up-regulation of CCL21 and CCR7 mRNA, as well as CCL21 protein levels during LV pressure overload, the pathogenic significance of this finding is unclear. Increased CCL21 levels have been reported in atherosclerosis both systemically and within the lesion [9], but whether this represents a pathogenic inflammatory response or compensatory beneficial mechanisms leading to enhanced exit of leukocytes from the lesion is still unclear. Herein we found that although CCR7 deficient mice exposed to AB showed only a mild degree of impaired myocardial function, these mice exhibited LV dilation, suggesting an impaired adaptive response to pressure overload in these mice. Although we were unable to demonstrate altered myocardial collagen content and MMP-2 and -9 activity three weeks after aortic banding, it is possible that this might be present at an earlier time-point, at least partly explaining the increased dilation seen in CCR7^−/−^ mice. We have previously reported myocardial dilatation in CXCR5^−/−^ mice following AB indicating a role in adaptive remodeling for the homeostatic chemokine CXCL13 [21]. It is possible that a similar effect could be attributed to CCL21/CCR7 interaction. However, although CCL21 has been implicated in beneficial remodeling processes within the kidney [22], the role of CCL21/CCR7 interaction in myocardial remodeling following LV pressure overload is at present unclear.

The current study has some limitations such as the inclusion of relatively few patients, and in particular controls. We also lack data in patients with asymptomatic AS. Moreover, the changes in echocardiographic variables in the CCR7 deficient mice were rather modest and we lack data on the molecular mechanisms for these findings. Furthermore, we were unable to carry out immunofluorescent staining for CCL21 in hypertrophied murine myocardium and to confirm the cellular localization of CCR7 in calcified human aortic valves by co-staining with relevant antibodies. Also, the lack of myocardial samples from different time points hampered the ability to elucidate the mechanisms underlying our findings in the experimental part of the study. Nonetheless, our studies, combining experiments in clinical and experimental LV pressure overload, suggest that CCL21/CCR7 interactions might be involved in the response to pressure overload secondary to AS.

## References

[1] Opie LH, Commerford PJ, Gersh BJ, Pfeffer MA (2006) Controversies in ventricular remodelling. Lancet 367: 356–367. 16443044 10.1016/S0140-6736(06)68074-4

[2] Ross J Jr, Braunwald E (1968) Aortic stenosis. Circulation 38: 61–67. 4894151 10.1161/01.cir.38.1s5.v-61

[3] Freeman RV, Otto CM (2005) Spectrum of calcific aortic valve disease: pathogenesis, disease progression, and treatment strategies. Circulation 111: 3316–3326. 15967862 10.1161/CIRCULATIONAHA.104.486738

[4] Akat K, Borggrefe M, Kaden JJ (2009) Aortic valve calcification: basic science to clinical practice. Heart 95: 616–623. 18632833 10.1136/hrt.2007.134783

[5] Rajamannan NM, Bonow RO, Rahimtoola SH (2007) Calcific aortic stenosis: an update. Nat Clin Pract Cardiovasc Med 4: 254–262. 17457349 10.1038/ncpcardio0827

[6] Forster R, Davalos-Misslitz AC, Rot A (2008) CCR7 and its ligands: balancing immunity and tolerance. Nat Rev Immunol 8: 362–371. 18379575 10.1038/nri2297

[7] Muller G, Hopken UE, Stein H, Lipp M (2002) Systemic immunoregulatory and pathogenic functions of homeostatic chemokine receptors. J Leukoc Biol 72: 1–8. 12101256

[8] Pierce EM, Carpenter K, Jakubzick C, Kunkel SL, Evanoff H, et al (2007) Idiopathic pulmonary fibrosis fibroblasts migrate and proliferate to CC chemokine ligand 21. Eur Respir J 29: 1082–1093. 17331965 10.1183/09031936.00122806

[9] Damas JK, Smith C, Oie E, Fevang B, Halvorsen B, et al (2007) Enhanced expression of the homeostatic chemokines CCL19 and CCL21 in clinical and experimental atherosclerosis: possible pathogenic role in plaque destabilization. Arterioscler Thromb VascBiol 27: 614–620. 10.1161/01.ATV.0000255581.38523.7c 17170367

[10] Kaur D, Saunders R, Berger P, Siddiqui S, Woodman L, et al (2006) Airway smooth muscle and mast cell-derived CC chemokine ligand 19 mediate airway smooth muscle migration in asthma. Am J Respir Crit Care Med 174: 1179–1188. 16959919 10.1164/rccm.200603-394OC

[11] Pickens SR, Chamberlain ND, Volin MV, Pope RM, Talarico NE, et al (2012) Role of the CCL21 and CCR7 pathways in rheumatoid arthritis angiogenesis. Arthritis Rheum 64: 2471–2481. 22392503 10.1002/art.34452 PMC3409328

[12] Kawashima D, Oshitani N, Jinno Y, Watanabe K, Nakamura S, et al (2005) Augmented expression of secondary lymphoid tissue chemokine and EBI1 ligand chemokine in Crohn's disease. J Clin Pathol 58: 1057–1063. 16189151 10.1136/jcp.2004.024828 PMC1770738

[13] Yndestad A, Finsen AV, Ueland T, Husberg C, Dahl CP, et al (2012) The homeostatic chemokine CCL21 predicts mortality and may play a pathogenic role in heart failure. PLoS One 7: e33038. 22427939 10.1371/journal.pone.0033038 PMC3299722

[14] Skjaerpe T, Hegrenaes L, Hatle L (1985) Noninvasive estimation of valve area in patients with aortic stenosis by Doppler ultrasound and two-dimensional echocardiography. Circulation 72: 810–818. 3896562 10.1161/01.cir.72.4.810

[15] Lang RM, Bierig M, Devereux RB, Flachskampf FA, Foster E, et al (2005) Recommendations for chamber quantification: a report from the American Society of Echocardiography's Guidelines and Standards Committee and the Chamber Quantification Writing Group, developed in conjunction with the European Association of Echocardiography, a branch of the European Society of Cardiology. J Am Soc Echocardiogr 18: 1440–1463. 16376782 10.1016/j.echo.2005.10.005

[16] Ngo DT, Wuttke RD, Turner S, Marwick TH, Horowitz JD (2004) Quantitative assessment of aortic sclerosis using ultrasonic backscatter. J Am Soc Echocardiogr 17: : 1123 1130. 10.1016/j.echo.2004.06.012 15502785

[17] Finsen AV, Christensen G, Sjaastad I (2005) Echocardiographic parameters discriminating myocardial infarction with pulmonary congestion from myocardial infarction without congestion in the mouse. Journal of Applied Physiology 98: 680–689. 15475595 10.1152/japplphysiol.00924.2004

[18] Liu H, Sanuda-Pena MC, Harvey-White JD, Kalra S, Cohen SA (1998) Determination of submicromolar concentrations of neurotransmitter amino acids by fluorescence detection using a modification of the 6-aminoquinolyl-N-hydroxysuccinimidyl carbamate method for amino acid analysis. J Chromatogr A 828: 383–395. 9916319 10.1016/s0021-9673(98)00836-x

[19] Laurent GJ, Cockerill P, McAnulty RJ, Hastings JR (1981) A simplified method for quantitation of the relative amounts of type I and type III collagen in small tissue samples. Anal Biochem 113: 301–312. 7283136 10.1016/0003-2697(81)90081-6

[20] Bruhl H, Mack M, Niedermeier M, Lochbaum D, Scholmerich J, et al (2008) Functional expression of the chemokine receptor CCR7 on fibroblast-like synoviocytes. Rheumatology(Oxford) 47: 1771–1774. 18838387 10.1093/rheumatology/ken383

[21] Waehre A, Halvorsen B, Yndestad A, Husberg C, Sjaastad I, et al (2011) Lack of chemokine signaling through CXCR5 causes increased mortality, ventricular dilatation and deranged matrix during cardiac pressure overload. PLoS One 6: e18668. 21533157 10.1371/journal.pone.0018668 PMC3078912

[22] Banas B, Wornle M, Merkle M, Gonzalez-Rubio M, Schmid H, et al (2004) Binding of the chemokine SLC/CCL21 to its receptor CCR7 increases adhesive properties of human mesangial cells. Kidney Int 66: 2256–2263. 15569314 10.1111/j.1523-1755.2004.66037.x

